# Reducing Mouse Anxiety during Handling: Effect of Experience with Handling Tunnels

**DOI:** 10.1371/journal.pone.0066401

**Published:** 2013-06-20

**Authors:** Kelly Gouveia, Jane L. Hurst

**Affiliations:** Institute of Integrative Biology, University of Liverpool, Neston, Cheshire, United Kingdom; Kent State University, United States of America

## Abstract

Handling stress is a well-recognised source of variation in animal studies that can also compromise the welfare of research animals. To reduce background variation and maximise welfare, methods that minimise handling stress should be developed and used wherever possible. Recent evidence has shown that handling mice by a familiar tunnel that is present in their home cage can minimise anxiety compared with standard tail handling. As yet, it is unclear whether a tunnel is required in each home cage to improve response to handling. We investigated the influence of prior experience with home tunnels among two common strains of laboratory mice: ICR(CD-1) and C57BL/6. We compared willingness to approach the handler and anxiety in an elevated plus maze test among mice picked up by the tail, by a home cage tunnel or by an external tunnel shared between cages. Willingness to interact with the handler was much greater for mice handled by a tunnel, even when this was unfamiliar, compared to mice picked up by the tail. Once habituated to handling, C57BL/6 mice were most interactive towards a familiar home tunnel, whereas the ICR strain showed strong interaction with all tunnel handling regardless of any experience of a home cage tunnel. Mice handled by a home cage or external tunnel showed less anxiety in an elevated plus maze than those picked up by the tail. This study shows that using a tunnel for routine handling reduces anxiety among mice compared to tail handling regardless of prior familiarity with tunnels. However, as home cage tunnels can further improve response to handling in some mice, we recommend that mice are handled with a tunnel provided in their home cage where possible as a simple practical method to minimise handling stress.

## Introduction

Handling stress is often pointed out as a potential source of unexplained variation within and between animal studies. This is because handling stress is known to influence both the behaviour and physiology of animals [1]–[4]. Yet, the need for research on how to overcome confounding effects of handling in experimental studies has been underemphasized. This is particularly important because it is impractical to standardise exactly when, how frequently or for how long animals are handled for routine maintenance and experimental manipulations between studies; and yet stressful experiences may have a major impact on the status and responses of experimental animals. A major complication of variability in the responses of research animals is that it implies an increase in the numbers required for experiments [5], [6], while important responses may be overshadowed by handling-induced stress and missed. As mice are the most common species used in animal research worldwide, particularly in biomedical studies, understanding how to minimise any strong stress responses to handling is a priority that could affect a very large number of studies. In addition to the impact on research outcomes, the influence of routine handling on the expression of anxiety behaviour also raises concern for the welfare of many millions of mice that are kept within animal facilities.

**Figure 1:**
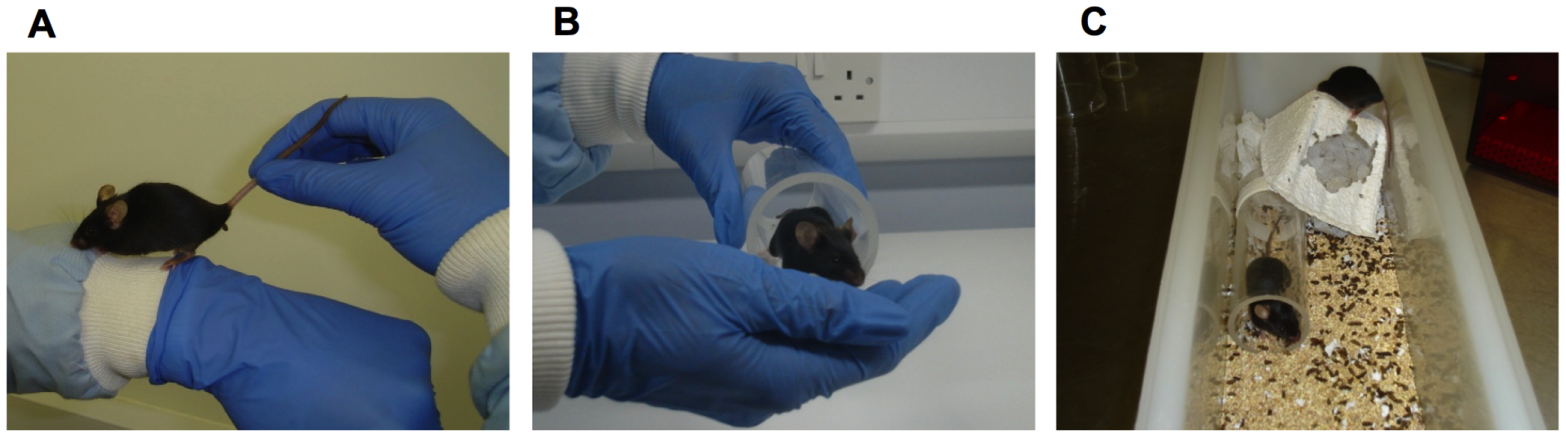
Handling methods used to pick up mice. (A) Tail handling: the most widespread method currently used for handling mice. The animal is lifted by the base of the tail between thumb and forefingers and supported on the handler’s arm or hand. (B) Tunnel handling. The animal is guided into a plastic tunnel and held inside the tunnel. (C) Tunnels may be provided in the home cage as source of enrichment for mice as well as a tool for handling.

The standard practice for handling mice is to pick them up by holding the base of the tail between thumb and forefingers. However, Hurst and West [7] found that this method induced greater anxiety than picking mice up in a home cage tunnel (Figure 1). Mice handled by their home tunnel were more willing to approach the handler than those picked up by the tail even after mice were then restrained by the scruff of the neck. They also showed lower anxiety in an elevated plus maze test. While this study showed a striking difference in response to handling by these different methods, it is unclear whether the reduced stress response to tunnel handling depends on mice being familiar with the handling tunnel. Providing a tunnel in every home cage may not be a feasible option for all animal units due to differences in husbandry practices across laboratories and the financial cost of home tunnels. An alternative may be to use an external tunnel that can be used for multiple cages if familiarity with the tunnel in the home cage is not important. This study thus aims to clarify the importance of providing tunnels in each home cage to tame the anxiety of mice to handling.

Common laboratory mouse strains are known to differ in their susceptibility to anxiety and neophobic responses [8]. For example, one of the most common inbred strains C57BL/6 shows greater anxiety when compared with the commonly used outbred ICR(CD-1) strain [7], [9]. It is essential that handling methods are robust to strain differences to minimise anxiety across strains. In this study, we compare response to different handling methods and tunnel experience using these two common laboratory strains, which have well established differences in their susceptibility to anxiety. We addressed: (i) whether handling mice by tunnel tamed anxiety to handling in comparison to tail handling even if mice were not provided with a home tunnel in their cage; (ii) if familiarity with a home tunnel influenced response to tunnel handling; (iii) the consistency of responses to handling across ICR (CD-1) and C57BL/6 mice of both sexes (referred to subsequently as ICR and C57 respectively).

## Results

To examine the effects of handling method and experience on anxiety-related responses, we assessed the amount of voluntary interaction with a handler immediately before and after handling, when the handler stood motionless with the handling device in the cage (gloved hand or gloved hand holding a handling tunnel). This allowed us to compare behaviour in anticipation of being handled between methods (tail or tunnel) that impose different constraints on the animal’s behaviour during the handling procedure itself, assessed during the first, fifth and ninth handling session. Assessment both immediately before and after handling in each session allowed us to examine the change in behaviour immediately after handling as well as longer term changes between sessions as animals became more familiar with handling. An increase or sustained high level of interaction after handling indicates a positive willingness to immediately return and interact with the handling device. After nine daily handling sessions of 60s handling per mouse to familiarise animals with a particular handling method, we also compared behaviour in an elevated plus maze test, a well established and validated test of anxiety in laboratory rodents [10]–[12]. No sex differences were found for any of the parameters addressed in this study and there were no interactions between sex and any of the other factors addressed (see Tables S1 and S2 in Supporting Information), so data are shown for males and females combined. Significant interactions between handling session (first, fifth and ninth) and all other factors (handling method, strain and before/after handling) indicated complex changes in response to the different methods over time between the two strains, so separate analyses compared response between handling methods within each separate handling session.

### Willingness to Interact with the Handler

#### Tail versus shared tunnel

**Figure 2:**
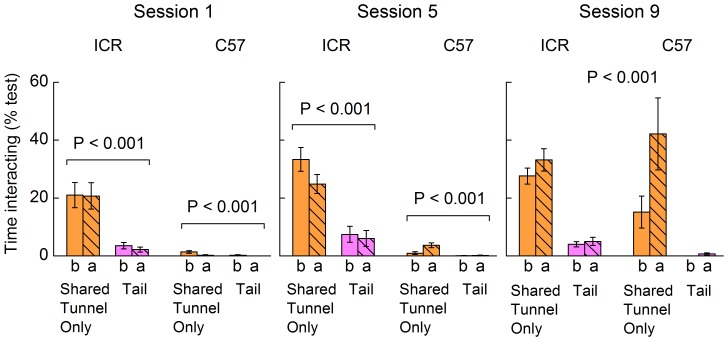
Voluntary interaction with the handler immediately before and after mice were picked up by a shared tunnel or tail. Percentage of test time interacting with the handler immediately before (b, solid bars) and after (a, hatched bars) the first, fifth and ninth handling session for C57 and ICR mice picked up by one of two different handling methods (mean ± s.e.m., n = 8 cages for each handling group and strain). Shared Tunnel Only and Tail mice were kept under the same housing conditions and neither had any prior experience of tunnels. P values indicate the effect of handling method (repeated measures ANOVAs, full analyses given in Table S1). There was a significant interaction between handling method and strain in session 1 (F_1,26_ = 22.2, P<0.001) and session 5 (F_1,26_ = 19.5, P<0.001) but not in session 9 (F_1,24_ = 0.1, P = 0.74).

We first established whether handling by a tunnel improves the willingness of mice to interact voluntarily with a handler compared to those handled by tail, even if they are not provided with a tunnel in their home cage. To assess this, one group of mice was handled by a shared tunnel (not present in the home cage) that was used for all mice of the same strain and sex, while the other group was handled by the tail. None of these mice had any prior experience of a home tunnel or of being handled using a tunnel. Overall, mice spent much more time in voluntary interaction with the handler when picked up by a shared tunnel than when handled by tail (Figure 2). However, strains differed in the number of handling sessions that it took to develop substantial voluntary interaction with a shared tunnel (method × strain interaction, session 1: F_1,26_ = 22.2, P<0.001; session 5: F_1,26_ = 19.5, P<0.001; session 9: F_1,24_ = 0.1, P = 0.74; Table S1). While the outbred ICR strain was willing to interact with the unfamiliar handling tunnel even on day one, C57 mice were only slightly more willing to interact with an unfamiliar handling tunnel than with tail handling when this was assessed on the first and fifth handling sessions. They showed a very high level of interaction with a shared tunnel equivalent to ICR mice only after nine handling sessions (Figure 2). By contrast, mice of both strains showed very little voluntary interaction when handled by tail, even after nine handling sessions (Figure 2).

Change in the willingness to interact immediately after handling compared to before depended on both handling method and strain (Table S1); separate analyses thus examined response to each method separately. Voluntary interaction immediately after tail handling did not differ from before, remaining very low in both strains across all handling sessions (Figure 2). When handled by a shared tunnel, ICR mice were just as willing to interact with an unfamiliar tunnel after their first experience of handling in session 1, indicating that the unfamiliar handling experience did not stimulate an immediate aversive response to the tunnel (Figure 2). By contrast, C57 mice were unwilling to interact initially with the tunnel with or without experience of being picked up in it, showing higher initial caution regardless of handling (effect of strain: F_1,14_ = 33.3, P<0.001; before/after handling: F_1,14_ = 0.06, P = 0.81). By session 9, when animals had become familiar with the shared handling tunnel, the change in response after handling differed slightly depending on strain (F_1,12_ = 6.03, P = 0.03). Both showed a similarly high willingness to interact with the tunnel immediately after handling, indicating a positive response to the now familiar experience of tunnel handling across strains, although C57 still showed a lower level of interaction with the tunnel before handling (but much higher than for tail handling) consistent with the slower development of a positive response towards the presence of a shared tunnel in this more cautious strain.

#### Experience of a handling tunnel in the home cage

**Figure 3:**
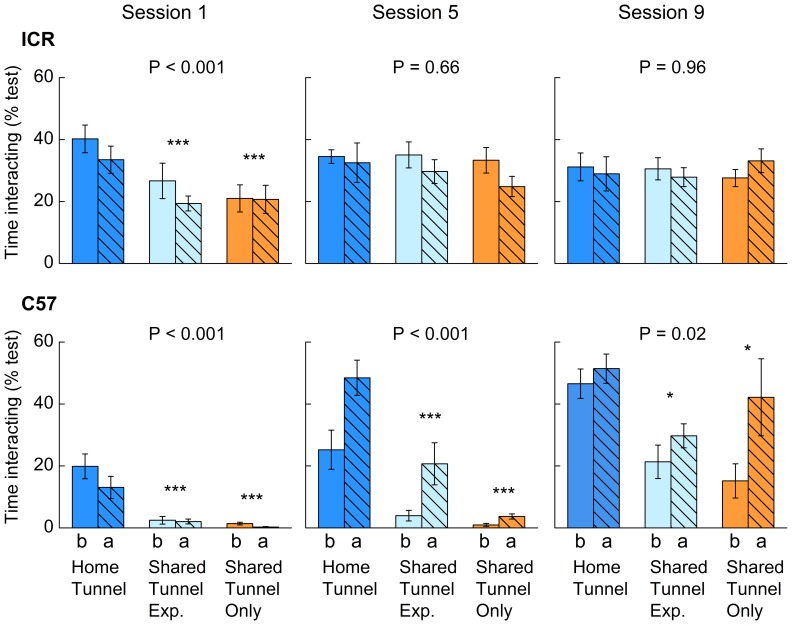
Effect of tunnel experience on voluntary interaction before and after handling by tunnel. Percentage of test time interacting with the handler immediately before (b, solid bars) and after (a, hatched bars) handling sessions one, five and nine for C57 and ICR mice handled either using a home tunnel or a shared tunnel (mean ± s.e.m., n = 8 cages per handling group for each strain). Mice handled with a shared tunnel either had prior experience with a home tunnel (Shared Tunnel Exp.) or no prior tunnel experience (Shared Tunnel Only). P values indicate the effect of tunnel experience for each strain (repeated measures ANOVAs, full analyses are given in Table S1). Asterisks indicate a significant planned contrast between the home tunnel method and shared tunnel groups (* P<0.05, ** P<0.01, ***P<0.001).

To establish whether familiarity with a tunnel in the home cage influences the response of mice to tunnel handling, we compared three groups: (1) mice handled by their familiar home cage tunnel (Home Tunnel), (2) mice handled by a shared tunnel (not present in the home cage) but after one week experience with a tunnel in the home cage (Shared Tunnel Experienced), or (3) mice handled by a shared tunnel (not present in the home cage) that had no prior contact with a tunnel (Shared Tunnel Only). This allowed us to assess the extent to which mice respond better when handled with a highly familiar tunnel kept permanently in the home cage compared to a shared tunnel encountered only during handling, and whether giving animals prior experience with a tunnel in their home cage for a limited period would improve response to a shared handling tunnel. Familiarity with the handling tunnel from the animal’s home cage significantly influenced the willingness of mice to interact with a handling tunnel during the first handling session (F_2,36_ = 17.7, P<0.001; Table S1). Mice spent longer interacting with a familiar home cage tunnel than with an unfamiliar shared tunnel that was not present in their home cage (Figure 3, session 1). This difference in response to familiar home cage versus unfamiliar shared handling tunnels was seen in both strains, with no interaction between tunnel type and strain (F_2,36_ = 0.005, P = 0.99), although ICR spent much longer than C57 mice in voluntary interaction with all handling tunnels in the first handling session (Figure 3). Response to an unfamiliar shared tunnel was very similar whether or not mice had prior experience with a tunnel in their home cage before the first handling session. Willingness to interact showed a slight decrease immediately after first handling compared to before across all methods and strains (F_1,36_ = 5.9, P = 0.02; Table S1). However, where animals were willing to interact strongly with tunnels before handling, interaction was only slightly less strong after handling (Figure 3, session 1). Thus, the first experience of being picked up and held in a tunnel did not substantially reduce willingness to interact with the handling device compared to the bigger differences between methods and strains.

Over repeated handling sessions, a strain difference in responsiveness to the different types of handling tunnel became apparent, with a significant interaction between tunnel handling group and strain in session 5 (F_2,36_ = 9.0, P = 0.001) and, to a lesser extent, in session 9 (F_2,34_ = 3.7, P = 0.04; Table S1). The strains also differed in how their response changed immediately after handling compared to beforehand (strain × before/after handling interaction, session 5: F_1,36_ = 27.4, P<0.001; session 9: F_1,34_ = 10.8, P = 0.002). These differences were because C57 mice continued to interact more with a home cage tunnel than with a shared handling tunnel (session 5: F_2,18_ = 21.7, P<0.001; session 9: F_2,15_ = 5.1, P = 0.02) both immediately before and after handling (Figure 3). C57 interaction was lowest towards shared tunnels when they had no prior experience of a tunnel in their home cage, but even in this situation their willingness to interact increased substantially over the nine handling sessions (F_1,17_ = 73.7, P<0.001). C57 willingness to interact with the tunnels was also elevated immediately after handling compared to beforehand in both sessions (session 5: F_1,21_ = 28.8, P<0.001; session 9: F_1,18_ = 13.5, P = 0.002), indicating that experience of being handled resulted in a positive reaction to all of the tunnels, but the positive response in session 5 was significantly stronger when C57 were previously familiar with tunnels and weakest when they were unfamiliar (Figure 3, method × before/after interaction: F_2,21_ = 5.23, P = 0.014). By contrast, ICR willingness to interact with shared tunnels was much more rapid such that they showed equally high interaction with home cage and shared handling tunnels after five (F _2,18_ = 0.4, P = 0.66) and nine sessions (F _2,19_ = 0.05, P = 0.96), regardless of prior experience with a tunnel in their home cage (Figure 3). They also showed the same high level of interaction immediately before and after handling regardless of handling method (session 5: F_1,21_ = 2.82, P = 0.11; session 9: F_1,22_ = 0.01, P = 0.93).

### Anxiety Behaviour

**Figure 4:**
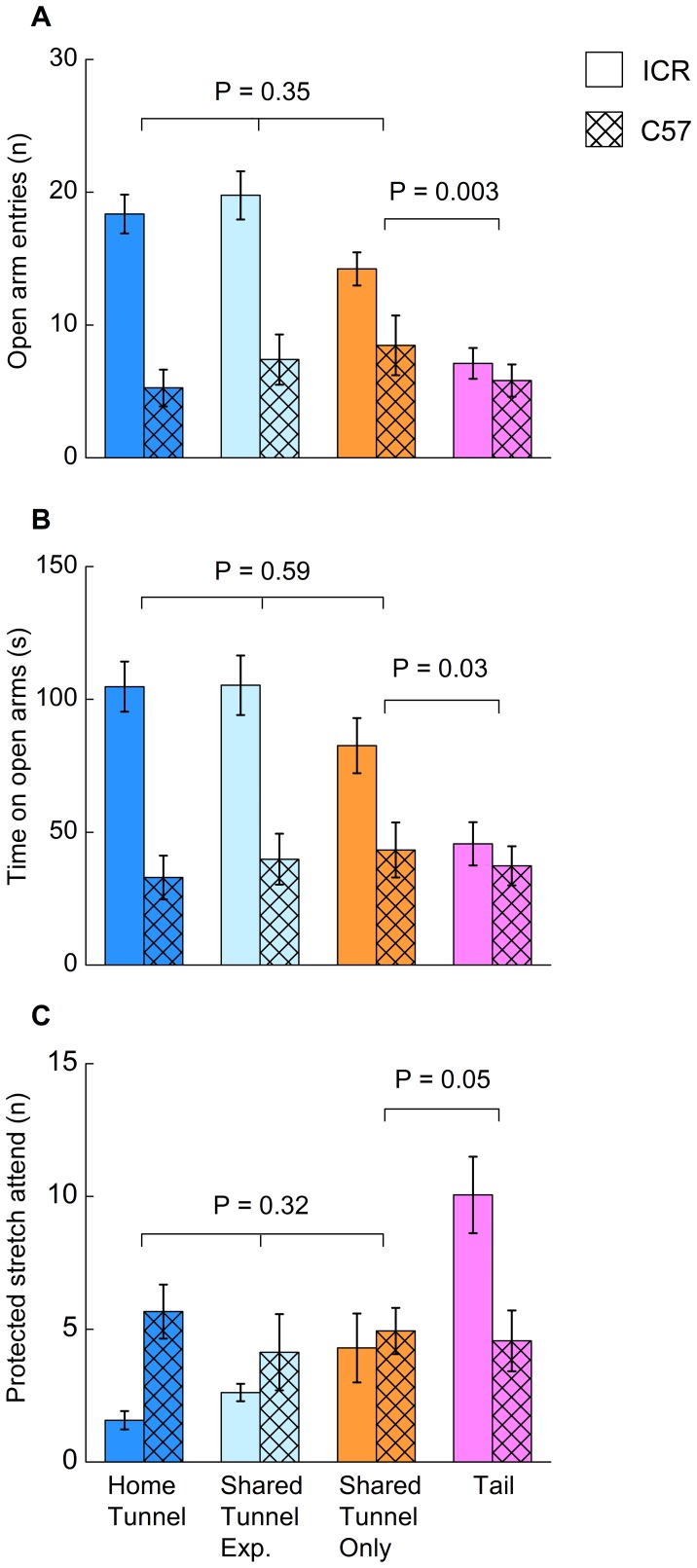
Effect of handling method and tunnel experience on anxiety measures. Frequency of open arm entries (A), time on open arms (B) and protected stretch attend postures (C) in an elevated plus maze test for ICR (open bars) and C57 (hatched bars) mice after nine sessions of handling by home tunnel, shared tunnel or tail (mean ± s.e.m., n = 16 mice per handling group). Mice handled with a shared tunnel either had prior experience with a home tunnel (Shared Tunnel Exp.) or no prior tunnel experience (Shared Tunnel Only). P values indicate the effect of tunnel experience between tunnel groups, or compared shared tunnel versus tail handling (one way ANOVAs, full analyses are given in Table S2). There were no significant interactions between method and strain, except for a difference in protected stretch attend between shared tunnel only and tail handled mice (F_1,57_ = 5.6, P = 0.02). Protected stretch attend was significantly higher among ICR mice handled by tail (P = 0.01) but not among C57 (P = 0.78).

Handling by a shared tunnel reduced anxiety behaviour in the elevated plus maze test compared to tail handling (full analyses are given in Table S2). Anxiety behaviour in this test is evident from a reluctance to visit the open arms of the maze and an increase in the exhibition of protected stretched attend postures, a type of risk assessment behaviour [11], [13]. The number of entries to the open arms (F_1,57_ = 9.9, P = 0.003) and total time spent on the open arms (F_1,57_ = 4.9, P = 0.03) were greater among those handled by a shared tunnel than by tail (Figure 4A,B). While there was no significant interaction between handling method and strain, differences in response to the open arms were much more pronounced in the ICR strain (Figure 4). ICR mice handled by a shared tunnel also exhibited fewer protected stretched attend postures than those handled by the tail (F_1,30_ = 7.5, P = 0.01), a difference that was not apparent among C57 mice (F_1,27_ = 0.08, P = 0.78; interaction between strain and handling method, F_1,57_ = 5.6, P = 0.02; Figure 4C).

Neither the type of tunnel used (home cage or shared), nor prior experience of a tunnel in the home cage, had any influence on anxiety measures among mice handled by tunnel in the elevated plus maze test. Compared to ICR mice, C57 mice handled by tunnel spent much less time on the open arms (F_1,76_ = 49.1, P<0.001), visited the open arms less frequently (F_1,76_ = 53.0, P<0.001) and displayed more protected stretch attend postures (F_1,76_ = 5.2, P = 0.03; Figure 4) whether they were handled by a home cage tunnel or a tunnel shared by other mice of their own strain.

## Discussion

Recent evidence suggests that handling mice by a tunnel that is present in the home cage causes much less aversion than tail handling [7]. Here we have shown that handling mice using a tunnel reduces anxiety in comparison to tail handling even when mice are unfamiliar with home tunnels. Thus, the substantial difference in response of mice to being picked up using a handling tunnel rather than by the tail does not rely on the use of a familiar home cage tunnel for handling.

Initially, familiarity with a handling tunnel that was present in the home cage improved voluntary interaction with the handler among both mouse strains tested. Mice that were handled by their familiar home tunnel spent more time interacting with the handler than those handled using an external shared tunnel. As temporary experience with a home tunnel did not improve this initial response to a shared tunnel, the reduced time spent interacting with a shared compared to a home cage tunnel was most likely due to caution in approaching its novel scent. However, once mice had been handled several times, differences in response to handling among these groups were less obvious because willingness to interact with a shared tunnel increased. Accordingly, after nine daily handling sessions, mice handled by home or shared tunnels showed similar reduced anxiety in the elevated plus maze. Indeed, it is known that habituating rodents to handling minimises aversion towards human contact [7], [14], [15]. This study further emphasizes the importance of repeated handling using a non-aversive method for taming anxiety-like responses of mice to handling, particularly when using a handling tunnel that is unfamiliar and thus provokes initial caution. However, by contrast, animals handled by the tail consistently avoided any interaction with the handler and failed to show habituation on repeated handling. These findings are in agreement with Hurst and West [7] and, together, indicate that repeated experience of tail handling, which induces strong aversion, stress and anxiety responses in mice, does not minimise negative responses towards human contact. Thus, habituating animals to handling requires the use of a method that does not induce strong negative responses in the animals, otherwise this might sensitize rather than decrease their stress on handling.

Female and male mice showed very similar responses to the different handling methods as previously found in the Hurst and West [7] study. Further, both strains showed the same general difference in response to tunnel and tail handling, emphasizing the robustness of these methods for handling mice of different strains and sexes. In comparison to ICR mice though, C57 mice showed a slower habituation to handling by a shared tunnel in comparison to handling by their home tunnel. This difference in behaviour between the two strains is likely to be due to strain-specific differences in emotional reactivity that have previously been identified [9], [16], [17]. This is corroborated by the greater anxiety displayed by C57 mice in the elevated plus maze compared to ICR mice. It would be interesting to explore the extent to which the anxiety displayed by C57 mice in the elevated plus maze might be reduced by additional experience of non-aversive handling; however, the behaviour of those handled repeatedly using a home cage tunnel to which they readily habituated suggests that this higher level of anxiety is not a response to handling stress.

Our results suggest that mouse studies could benefit from refining the method of handling used during experimental testing and during routine husbandry. By contrast to tail handling mice do not show avoidance of handling when picked up by a tunnel and anxiety is lower. Anxiety responses associated with handling and any subsequent variability due to changes in behavioural and physiological status induced by handling are likely to be considerably reduced by handling mice using a tunnel. Although handling cannot be avoided in the laboratory and is impossible to equate between studies from a practical perspective, minimising anxiety-like responses could help improve the robustness of research that uses mice. Ultimately, reducing handling-induced anxiety as a source of variation in experimental research contributes towards a reduction in the number of animals required for experiments whilst potentially also increasing the ability to detect subtle responses. As mice are handled frequently throughout their lives during routine husbandry and experimental procedures, least aversive handling will also contribute to improve the welfare of mice kept in animal facilities.

The findings from this study have important implications for the practical implementation of handling that is least aversive to mice. We have shown that anxiety associated with handling may be reduced if mice are picked up by a shared tunnel rather than by the tail. A shared tunnel could therefore be a viable option to refine handling when tunnels are not provided in each home cage. In this study we used a different shared tunnel for each sex. Although mice are attracted to scents of the opposite sex [18], [19] which might improve willingness to enter shared handling tunnels even further, scents from the opposite sex can have important priming effects on behaviour and physiology that could have confounding effects. For example, exposure of males to female scents increases androgen production, stimulating faster maturation and increasing the aggressiveness of adult males, which could cause problems within male groups. However, exposure to 2,5-dimethylpyrazine which is produced at high levels by group-housed females can suppress maturation of male reproductive organs and may also suppress immunocompetence (reviewed in [20]). Contact with male scents generally stimulates female reproductive physiology, bringing females into oestrus (reviewed in [20]) but uncontrolled exposure to male scents during handling could result in increased variation in female physiological status according to the amount and timing of individual experience. Contact with unfamiliar male urine can also block pregnancy in newly conceived females [21] and thus would be a particular problem if shared tunnels containing male scents were used for breeding females. Thus, we advise that shared handling tunnels are not shared between sexes to avoid such problems. However, the main advantage of using a home tunnel instead of a shared tunnel is that very little handling is required for mice to show habituation and to develop a strongly positive approach towards these tunnels. For this reason, we recommend that home tunnels are provided to all home cages as a more practical means of handling mice via tunnel. Home tunnels also have additional benefits for mouse welfare as a source of enrichment in the home cage, allowing animals greater control over their environment which in turn may decrease emotionality [22], [23]. Several features of the tunnels used here and by Hurst and West [7] enhance the practicality of tunnel handling. A smooth plastic material rather than alternatives such as cardboard provides the advantage that mice are unable to grip the inside of the tunnel and can be tipped out easily onto the hand or back into the cage after handling (gently tipping animals out backwards onto the surface works best). Use of a highly transparent tunnel also allows clear visual inspection of animals within the tunnel, allowing the handler to check that appearance, posture and movement is normal. This is an advantage over tail handling where this physical manipulation conflicts with normal posture and movement. The length (15 cm) and diameter (5 cm) of the tunnels also allowed sufficient space for animals to enter and move around freely without easily falling from the tunnel ends, as mice may more readily fall from shorter tunnels and can be more reluctant to enter much narrower tunnels. As handling mice by a tunnel requires minimal physical contact with the animal, this is likely to be another advantage for less experienced handlers. However, although human contact with the animal is minimised during tunnel handling, this does not induce aversion towards approach of the handler or direct physical contact as shown in the present study and by Hurst and West (2010).

### Conclusions

Handling mice by a shared tunnel that is not present in the home cage reduces anxiety and increases willingness to voluntarily approach the handler when compared with standard tail handling. The use of tunnels present in the home cage may further improve response to handling among more anxious strains. Therefore, to ensure mouse welfare during handling we recommend that mice are picked up by a tunnel and that where possible the handling tunnel is provided in each home cage for both handling and enrichment.

## Methods

### Ethics Statement

All procedures involved in this study were non-invasive behavioural tests and standard husbandry and handling procedures that did not involve any pain, suffering, distress or lasting harm. Instead, our study was designed to assess whether the standard handling method widely used for laboratory mice could be improved to reduce any negative responses to routine handling. Our work followed national and international best practice guidelines and was approved by the University of Liverpool Animal Welfare Committee but no specific licenses were required to carry out the work.

### Animals and Housing

Subjects were male and female mice of two common laboratory strains: C57BL/6JOlaHsd and ICR(CD-1) (n = 64 mice of each strain). ICR mice were obtained from an approved supplier (Harlan UK) at 3–4 weeks of age whereas C57 mice were bred in house (parents obtained from Harlan UK). At 3–4 weeks of age the mice were housed in single sex pairs in 43 × 11.5 × 12 cm cages (M3, North Kent Plastics Rochester, UK) and the pairs were maintained throughout the study. Bedding consisted of Corn Cob Absorb 10/14 substrate and paper wool was used as nest material. Water and food (lab diet 5002 certified rodent diet, Purina Mills) were given *ad-libitum*. Animals were housed under a reversed 12h light/dark schedule (lights on 8pm–8am). Cages were cleaned once every fortnight prior to the two week test period, with mice handled by the tail by experienced animal care staff to transfer them between cages.

To address the effect of prior familiarity with home tunnels on the response of mice to handling, we compared voluntary interaction with the handler and anxiety among mice assigned to one of three handling groups (n = 8×2 mice in each group for each strain): (i) handled using a tunnel present in the home cage (Home Tunnel); (ii) handled using an external tunnel shared between cages following experience with a tunnel in the home cage for one week before handling began (Shared Tunnel Experienced) (iii) handled with an external shared tunnel but with no experience of a home cage tunnel (Shared Tunnel Only). Shared tunnels were kept within the room and used exclusively for handling mice of the same strain and sex. To assess whether handling mice by a shared tunnel improved response to handling in comparison to tail handling, we compared handler interaction and anxiety between the Shared Tunnel Only group and a fourth group handled by the tail (neither having any experience with a home tunnel). Home and shared handling tunnels were transparent acrylic hollow cylinders, measuring 50 mm wide and 150 mm long. Home Tunnel and Shared Tunnel Experienced groups both received home tunnels ten days prior to testing but these were removed from the Shared Tunnel Experienced group after seven days. All animals were individually marked using hair dye on the shoulder or rump regions using Clairol Nice and Easy Natural Black® for ICR mice and Jerome Russell B-Blonde® for C57 mice three days prior to testing. Fur dyeing was the preferred method of identification as previous pilot work showed no interference of this technique on the response of mice to handling. For marking, animals were picked up and restrained by the tail on top of an empty MB1 cage 45×28×13 cm (North Kent Plastics, Rochester, UK). Once the animal was restrained the dye was applied using a small paint brush. The animal was then delivered back to its home cage and left undisturbed for 20 minutes to enable sufficient time for activation of the hair dye. Hair dye was washed off by gently applying wet cotton wool (soaked in warm tap water) against the animal’s fur. The marked area was gently dried off with cotton wool. During fur dye application and wash off procedures all animals were held for less than one minute to minimise handling stress.

Throughout the study, all handling procedures were carried out by the same experimenter along with transcription of behavioural data from DVD recordings. It was not possible to blind handling treatments as this required the use of physically different procedures. However, the differences found previously in response to tail and home cage tunnel handling are not subtle [7] and thus extremely unlikely to be due to any unconscious handler or observer bias. Responses (including strain differences) to these two handling treatments in the current study were also highly consistent with those found by independent handlers and observers in the earlier studies reported by Hurst & West [7], confirming repeatability of the strong differences in response to these two different handling methods.

### Handling Sessions

Handling sessions began when mice were 7–8 weeks old. The mice were handled once daily throughout nine sessions. Before handling the nest material and home tunnel were removed from the cage to allow assessment of voluntary interaction (when mice could not stay asleep in their nest) and to facilitate standard handling procedures. Nest material was carefully manipulated to avoid damaging the structure of the nest during handling sessions. For tunnel handling, the tunnel was brought towards the animal and held resting on the cage substrate while the animal was guided towards it with the other hand. Hands were cupped loosely over the tunnel ends to prevent escape until the mice were habituated to handling. Tail handling consisted of grasping the tail base between the thumb and forefingers, gently lifting the animal onto the experimenter’s gloved hand or forearm. Animals were then held in the tunnel or on the experimenter’s hand (tail) for 30 s. After handling both mice in the cage, the experimenter moved away from the cage for 60 s before handling the mice a second time so that each mouse was held for a total of 60 s per session. The handler wore a laboratory coat that was contaminated with mouse scent from previous handling sessions and clean close fit nitrile gloves that were rubbed in soiled bedding (from animals of same sex and strain) prior to the start of each handling session and whenever handling mice of the opposite sex. Tunnels were wiped clean with paper towel if contaminated by the animals during handling. The order in which cages were handled was balanced across handling sessions and handling treatments. Handling sessions were conducted within the first half of the dark phase of the light cycle under red lights to minimise variation due to differences in activity levels.

### Interaction Tests

To measure the willingness of mice to approach the experimenter in anticipation of handling, voluntary interaction with the handler was assessed 60 s immediately before and after the first, fifth and ninth handling sessions (as described in Hurst & West, [7]). Once the nest material and tunnel (if present) was removed from the home cage, the handler stood motionless facing the front of the cage for 60 s. A gloved hand (tail group) or gloved hand holding a tunnel (home or shared tunnel according to group) was held resting on the substrate in the front half of the cage without moving for 60 s. Following interaction testing the animals were handled by their designated method, after which the experimenter stood back from the cage for 60 s and then carried out a second interaction test. The durations of the following behaviours were recorded as voluntary interaction with the handler: sniffing (nose within 0.5 cm of the handling device), inside tunnel (all four paws inside), climbing (all four paws on handling device), paw contact (front paws on handling device), peeking in tunnel (animal places front paws in tunnel and immediately retreats from tunnel).

### Anxiety Testing

On the day following the last handling session mice were tested in an elevated plus maze. The test arena consisted of a white opaque plastic maze consisting of two open and two closed arms (all 30×5 cm with side walls 15 cm high on the two closed arms) elevated 57 cm above the ground. The mice were delivered to the centre of the arena, facing an open arm for a 5 min test. The total time and number of entries to each arm (closed and open), and protected stretch attend postures extending into the open arms from the central hub or closed arms were scored. At the end of testing the mice were returned to their home cage by their assigned handling method and the arena was wiped clean using a J cloth soaked in Teepol® detergent and dried clean with a paper towel. All mice were tested during the dark phase of the light cycle under red lights.

### Data Collection and Analysis

All observations were recorded remotely on DVD to a video recorder that was set up in the animal room. The recordings were subsequently analysed using customized timer software developed at the University of Liverpool (RJ Beynon). Statistical analysis was completed using SPSS 20.0 Graduate Pack statistical analysis software. The duration of each behaviour during interaction testing was averaged for both animals in the same cage as cagemates were unlikely to behave independently. The measures of voluntary approach were summed and expressed as the percentage of total test time interacting with the handler for analyses. Graphical inspection and Shapiro-Wilk tests determined normality distribution of the variables tested. Repeated measures ANOVAs compared interaction in each session (first, fifth, ninth) immediately before and after handling as within subject factors with tunnel experience, handling method (tail versus tunnel or three tunnel groups), strain and sex as between subjects effects. As this revealed significant interactions (P<0.05) between handling session and each of the other factors in the analysis except for sex (for the analyses of both tail versus shared tunnel and for the three tunnel groups), separate ANOVAs were conducted for each handling session. Analyses were further broken down to examine other interaction effects. Univariate ANOVAs examined the effects of handling method (tail *vs* tunnel or three tunnel groups) on responses in the elevated plus maze including sex and strain as additional factors. Planned contrasts compared Home Tunnel mice with Shared Tunnel Experienced and Shared Tunnel Only mice. All raw data used in these analyses are freely available from the authors on request.

## Supporting Information

Table S1 **Effects of tunnel experience, handling method, strain and sex on voluntary interaction with the handler immediately before and after handling (repeated measures ANOVAs).**
(DOCX)

Table S2 **Effects of tunnel experience, handling method, strain and sex on anxiety (a) and non-anxiety measures (b) in an elevated plus maze test (EPM).**
(DOCX)

## References

[1] Meaney MJ, Diorio J, Francis D, Widdowson J, LaPlante P, et al (1996) Early environmental regulation of forebrain glucocorticoid receptor gene expression: Implications for adrenocortical responses to stress. Dev Neurosci 18: 49–72. 8840086 10.1159/000111395

[2] Núñez JF, Ferré P, Escorihuela RM, Tobeña A, Fernández-Teruel A (1996) Effects of Postnatal Handling of Rats on Emotional, HPA-Axis, and Prolactin Reactivity to Novelty and Conflict. Physiol Behav 60: 1355–1359. 8916194 10.1016/s0031-9384(96)00225-9

[3] Balbcombe JP, Barnard ND, Sandusky C (2004) Laboratory routines cause animal stress. Contemp Top Lab Anim Sci 43: 42–51. 15669134

[4] Meijer MK, Sommer R, Spruijt BM, van Zutphen LF, Baumans V (2007) Influence of environmental enrichment and handling on the acute stress response in individually housed mice. Lab Anim 41: 161–173. 17430616 10.1258/002367707780378168

[5] Festing MWF, Baumans V, Combes RD, Halder M, Hendriksen CFM, et al (1998) Reducing the Use of Laboratory Animals in Biomedical Research: Problems and Possible Solutions. Altern Lab Anim 26: 283–301. 26042346

[6] Howard BR (2002) Control of variability. ILAR J 43: 194–201. 12391394 10.1093/ilar.43.4.194

[7] Hurst JL, West R (2010) Taming anxiety in mice by non-aversive handling. Nat Methods 7: 825–826. 20835246 10.1038/nmeth.1500

[8] Kim S, Lee S, Ryu S, Suk J, Park C (2002) Comparative analysis of the anxiety-related behaviors in four inbred mice. Behav Processes 60: 181–190. 12426069 10.1016/s0376-6357(02)00085-2

[9] Michalikova S, van Rensburg R, Chazot PL, Ennaceur A (2010) Anxiety responses in Balb/c, c57 and CD-1 mice exposed to a novel open space test. Behav Brain Res 207: 402–417. 19900487 10.1016/j.bbr.2009.10.028

[10] Lister RG (1987) The use of a plus-maze to measure anxiety in the mouse. Psychopharmacology. 92: 180–185. 10.1007/BF00177912 3110839

[11] Rodgers RJ, Dalvi A (1997) Anxiety, defence and the elevated plus-maze. Neurosci Biobehav Rev 21: 801–810. 9415905 10.1016/s0149-7634(96)00058-9

[12] Walf AA, Frye CA (2007) The use of the elevated plus maze as an assay of anxiety-related behavior in rodents. Nat. Protocols 2: 322–328. 17406592 10.1038/nprot.2007.44 PMC3623971

[13] Pellow S, Chopin P, File SE, Briley M (1985) Validation of open: closed arm entries in an elevated plus-maze as a measure of anxiety in the rat. J Neurosci Methods 14: 149–67. 2864480 10.1016/0165-0270(85)90031-7

[14] Roy V, Chapillon P (2004) Further evidences that risk assessment and object exploration behaviours are useful to evaluate emotional reactivity in rodents. Behav Brain Res 154: 439–448. 15313032 10.1016/j.bbr.2004.03.010

[15] Maurer BM, Döring D, Scheipl F, Küchenhoff H, Erhard MH (2008) Effects of a gentling programme on the behaviour of laboratory rats towards humans. Appl Anim Behav Sci 114: 554–571.

[16] Parmigiani S, Palanza P, Rodgers J, Ferrari PF (1999) Selection, evolution of behavior and animal models in behavioral neuroscience. Neurosci Biobehav Rev 23: 957–970. 10580310 10.1016/s0149-7634(99)00029-9

[17] Nicol CJ, Brocklebank S, Mendl M, Sherwin CM (2008) A targeted approach to developing environmental enrichment for two strains of laboratory mice. Appl Anim Behav Sci 110: 341–353.

[18] Moncho-Bogani J, Lanuza E, Hernández A, Novejarque A, Martínez-García F (2002) Attractive properties of sexual pheromones in mice: Innate or learned? Physiol Behav 77: 167–176. 12213516 10.1016/s0031-9384(02)00842-9

[19] Pankevitch DE, Cherry JA, Baum MJ (2006) Effect of vomeronasal organ removal from male mice on their preference for and neural fos responses to female urinary odors. Behav Neurosci 120: 925–936. 16893298 10.1037/0735-7044.120.4.925 PMC2263134

[20] Koyama S (2004) Primer effects by conspecific odors in house mice: a new perspective in the study of primer effects on reproductive activities. Hormon Behav 46: 303–310. 10.1016/j.yhbeh.2004.03.002 15325230

[21] Bruce HM (1959) An exteroceptive block to pregnancy in the mouse. Nature 184: 105. 13805128 10.1038/184105a0

[22] Wiepkema PR, Koolhaas JM (1993) Stress and animal welfare. Anim Welf 2: 195–218.

[23] Olsson AIS, Dahlborn K (2002) Improving housing conditions for laboratory mice: a review of environmental enrichment. Lab Anim 36: 243–270. 12144738 10.1258/002367702320162379

