# Supplementary material for: Reducing Mouse Anxiety during Handling: Effect of Experience with Handling Tunnels
Source: PLoS One. 2013 Jun 20;8(6):e66401. doi: 10.1371/journal.pone.0066401 (PMC3688777; doi:10.1371/journal.pone.0066401)
Supplement: Table S1 — Effects of tunnel experience, handling method, strain and sex on voluntary interaction with the handler immediately before and after handling (repeated measures ANOVAs). (DOCX) [file pone.0066401.s001.docx]

| **Voluntary interaction** | **Handling session 1** | | **Handling session 5** | | **Handling session 9** | |
| --- | --- | --- | --- | --- | --- | --- |
| *Tunnel experience* |  |  |  |  |  |  |
| Method | F_2,36_ = 17.7 | P < 0.001 | F_2,36_ = 14.4 | P < 0.001 | F_2,34_ = 4.4 | P = 0.02 |
| Before/after | F_1,36_ = 5.9 | P = 0.02 | F_1,36_ = 6.5 | P = 0.02 | F_1,34_ = 12.2 | P = 0.001 |
| Strain | F_1,36_ = 70.8 | P < 0.001 | F_1,36_ = 22.2 | P < 0.001 | F_1,34_ = 1.2 | P = 0.27 |
| Sex | F_1,36_ = 0.04 | P = 0.90 | F_1,36_ = 0.8 | P = 0.37 | F_1,34_ = 0.03 | P = 0.86 |
| Method x before/after | F_2,36_ = 1.2 | P = 0.31 | F_2,36_ = 5.1 | P = 0.01 | F_2,34_ = 4.9 | P = 0.01 |
| Method x strain | F_2,36_ = 0.005 | P = 0.99 | F_2,36_ = 9.0 | P = 0.001 | F_2,34_ = 3.7 | P = 0.04 |
| Before/after x strain | F_1,36_ = 0.3 | P = 0.60 | F_1,36_ = 27.4 | P < 0.001 | F_1,34_ = 10.8 | P = 0.002 |
| Method x sex | F_2,36_ = 1.6 | P = 0.22 | F_2,36_ = 0.2 | P = 0.79 | F_2,34_ = 0.03 | P = 0.97 |
| Method x strain x sex | F_2,36_ = 1.04 | P = 0.36 | F_2,36_ = 1.2 | P = 0.31 | F_2,34_ = 0.7 | P = 0.49 |
| Planned contrasts**†** |  |  |  |  |  |  |
| Home Tunnel vs Shared Tunnel Experienced |  | P < 0.001 | C57Bl/6 only | P < 0.001 | C57Bl/6 only | P = 0.01 |
| Home Tunnel vs Shared Tunnel Only |  | P < 0.001 | C57Bl/6 only | P < 0.001 | C57Bl/6 only | P = 0.03 |
| Method* |  |  |  |  |  |  |
| ICR(CD1) |  |  | F _2,18_ = 0.4 | P = 0.66 | F _2,19_ = 0.05 | P = 0.96 |
| C57BL/6 |  |  | F _2,18_ = 21.7 | P < 0.001 | F _2,15_ = 5.1 | P = 0.02 |
| *Tail vs Shared Tunnel* |  |  |  |  |  |  |
| Method | F_1,26_ = 25.8 | P < 0.001 | F_1,26_ = 29.2 | P < 0.001 | F_1,24_ = 50.5 | P < 0.001 |
| Before/after | F_1,26_ = 0.3 | P = 0.56 | F_1,26_ = 4.5 | P = 0.04 | F_1,24_ = 20.9 | P < 0.001 |
| Strain | F_1,26_ = 37.6 | P < 0.001 | F_1,26_ = 55.5 | P < 0.001 | F_1,24_ = 0.6 | P = 0.44 |
| Sex | F_1,26_ = 0.0 | P = 0.98 | F_1,26_ = 0.5 | P = 0.49 | F_1,24_ = 0.01 | P = 0.92 |
| Method x before/after | F_1,26_ = 0.003 | P = 0.96 | F_1,26_ = 1.6 | P = 0.22 | F_1,24_ = 17.3 | P < 0.001 |
| Method x strain | F_1,26_ = 22.2 | P < 0.001 | F_1,26_ = 19.5 | P < 0.001 | F_1,24_ = 0.1 | P = 0.74 |
| Before/after x strain | F_1,26_ = 0.002 | P = 0.96 | F_1,26_ = 13.7 | P = 0.001 | F_1,24_ = 8.1 | P = 0.009 |
| Method x sex | F_1,26_ = 0.2 | P = 0.66 | F_1,26_ = 0.009 | P = 0.93 | F_1,24_ = 0.003 | P = 0.95 |
| Method x strain x sex | F_1,26_ = 0.2 | P = 0.62 | F_1,26_ = 0.009 | P = 0.93 | F_1,24_ = 0.3 | P = 0.61 |
| Method* |  |  |  |  |  |  |
| ICR (CD1) | F_1,15_ = 28.0 | P < 0.001 | F_1,15_ = 28.4 | P < 0.001 |  |  |
| C57Bl/6 | F_1,15_ = 5.9 | P = 0.03 | F_1,15_ = 21.0 | P < 0.001 |  |  |

**Table S1.** Effects of tunnel experience, handling method, strain and sex on voluntary interaction with the handler immediately before and after handling (repeated measures ANOVAs)

Voluntary interaction was measured immediately before and after handling sessions one, five and nine by a 60s interaction test.

n = 32 x 2 mice per strain; n = 8 x 2 mice per handling group for each strain; n = 4 x 2 mice per sex per handling group for each strain;

**†** Planned contrasts compare home tunnel method to shared tunnel groups.

* Where there is a significant strain x method interaction, the effect of method is examined separately for each strain
