# Supplementary material for: Reducing Mouse Anxiety during Handling: Effect of Experience with Handling Tunnels
Source: PLoS One. 2013 Jun 20;8(6):e66401. doi: 10.1371/journal.pone.0066401 (PMC3688777; doi:10.1371/journal.pone.0066401)
Supplement: Table S2 — Effects of tunnel experience, handling method, strain and sex on anxiety (a) and non-anxiety measures (b) in an elevated plus maze test (EPM). (DOCX) [file pone.0066401.s002.docx]

**Table S2.** Effects of tunnel experience, handling method, strain and sex on anxiety (a) and non-anxiety measures (b) in an elevated plus maze test (EPM).

| **EPM measures** | | | | | | | | | | |
| --- | --- | --- | --- | --- | --- | --- | --- | --- | --- | --- |
|  | **(a) Anxiety measures** | | | | | | **(b) Non-anxiety control measures** | | | |
|  | Protected stretch attend | | Time on open arms | | Open arm entries | | Time in closed arms | | Closed arm entries | |
|  | Test statistic | Significance | Test statistic | Significance | Test statistic | Significance | Test statistic | Significance | Test statistic | Significance |
| *Tunnel experience* |  |  |  |  |  |  |  |  |  |  |
| Method | F_2,76_ = 1.1 | P = 0.32 | F_2,76_ = 0.5 | P = 0.59 | F_2,76_ = 1.1 | P = 0.35 | F_2,76_ = 0.08 | P = 0.92 | F_2,76_ = 0.6 | P = 0.58 |
| Strain | F_1,76_ = 5.2 | P = 0.03 | F_1,76_ = 49.1 | P < 0.001 | F_1,76_ = 53.0 | P < 0.001 | F_1,76_ = 10.6 | P = 0.002 | F_1,76_ = 3.0 | P = 0.09 |
| Sex | F_1,76_ = 0.3 | P = 0.60 | F_1,76_ = 1.2 | P = 0.27 | F_1,76_ = 1.0 | P = 0.33 | F_1,76_ = 0.01 | P = 0.91 | F_1,76_ = 0.1 | P = 0.70 |
| Method x strain | F_2,76_ = 1.7 | P = 0.20 | F_2,76_ = 1.3 | P = 0.29 | F_2,76_ = 2.5 | P = 0.09 | F_2,76_ = 1.0 | P = 0.38 | F_2,76_ = 0.4 | P = 0.67 |
| Method x sex | F_2,76_ = 0.4 | P = 0.68 | F_2,76_ = 0.3 | P = 0.73 | F_2,76_ = 0.5 | P = 0.62 | F_2,76_ = 0.8 | P = 0.43 | F_2,76_ = 0.6 | P = 0.56 |
| Method x strain x sex | F_2,76_ =0.1 | P = 0.88 | F_2,76_ = 0.4 | P = 0.64 | F_2,76_ = 0.7 | P = 0.52 | F_2,76_ = 0.3 | P = 0.76 | F_2,76_ = 1.2 | P = 0.31 |
| *Tail vs Shared Tunnel* |  |  |  |  |  |  |  |  |  |  |
| Method | F_1,57_ = 4.1 | P = 0.05 | F_1,57_ = 4.9 | P = 0.03 | F_1,57_ = 9.9 | P = 0.003 | F_1,57_ = 5.3 | P = 0.03 | F_1,57_ = 0.2 | P = 0.67 |
| Strain | F_1,57_ = 3.5 | P = 0.07 | F_1,57_ = 8.6 | P = 0.005 | F_1,57_ = 7.4 | P = 0.009 | F_1,57_ = 3.8 | P = 0.06 | F_1,57_ = 3.6 | P = 0.06 |
| Sex | F_1,57_ = 0.1 | P = 0.91 | F_1,57_ = 3.7 | P = 0.06 | F_1,57_ = 3.6 | P = 0.07 | F_1,57_ = 1.8 | P = 0.19 | F_1,57_ = 0.7 | P = 0.42 |
| Method x strain | F_1,57_ = 5.6 | P = 0.02 | F_1,57_ = 2.4 | P = 0.13 | F_1,57_ = 1.8 | P = 0.19 | F_1,57_ = 0.2 | P = 0.64 | F_1,57_ = 0.3 | P = 0.56 |
| Method x sex | F_1,57_ = 0.01 | P = 0.92 | F_1,57_ = 0.0 | P = 0.98 | F_1,57_ = 0.1 | P = 0.75 | F_1,57_ = 0.01 | P = 0.91 | F_1,57_ = 0.9 | P = 0.35 |
| Method x strain x sex | F_1,57_ = 1.3 | P = 0.26 | F_1,57_ = 0.23 | P = 0.63 | F_1,57_ = 0.03 | P = 0.87 | F_1,57_ = 0.4 | P = 0.52 | F_1,57_ = 1.9 | P = 0.17 |
| Method* |  |  |  |  |  |  |  |  |  |  |
| ICR (CD1) | F_1,30_ = 7.5 | P = 0.01 |  |  |  |  |  |  |  |  |
| C57Bl/6 | F_1,27_ = 0.08 | P = 0.78 |  |  |  |  |  |  |  |  |

Mice were tested in an elevated plus maze after nine daily handling sessions.

Statistics give F ratios for one way ANOVAs.

n = 32 x 2 mice per strain; n = 8 x 2 mice per handling group for each strain; n = 4 x 2 mice per sex per handling group for each strain;

* Where there is a significant strain x method interaction, the effect of method is examined separately for each strain
